# Compositions and Co-occurrence Patterns of Bacterial Communities Associated With Polymer- and ASP-Flooded Petroleum Reservoir Blocks

**DOI:** 10.3389/fmicb.2020.580363

**Published:** 2020-12-01

**Authors:** Guoling Ren, Jinlong Wang, Lina Qu, Wei Li, Min Hu, Lihong Bian, Yiting Zhang, Jianjun Le, Xumou Dou, Xinhong Chen, Lulu Bai, Yue Li

**Affiliations:** ^1^Heilongjiang Provincial Key Laboratory of Oilfield Applied Chemistry and Technology, Daqing, China; ^2^College of Bioengineering, Daqing Normal University, Daqing, China; ^3^Exploration and Development Research Institute, Daqing Oil Field Company, Ltd., Daqing, China

**Keywords:** bacterial community composition, network analysis, Illumina MiSeq sequencing, polymer-flooding, ASP-flooding

## Abstract

Polymer flooding technology and alkaline-surfactant-polymer (ASP) flooding technology have been widely used in some oil reservoirs. About 50% of remaining oil is trapped, however, in polymer-flooded and ASP-flooded reservoirs. How to further improve oil recovery of these reservoirs after chemical flooding is technically challenging. Microbial enhanced oil recovery (MEOR) technology is a promising alternative technology. However, the bacterial communities in the polymer-flooded and ASP-flooded reservoirs have rarely been investigated. We investigated the distribution and co-occurrence patterns of bacterial communities in ASP-flooded and polymer-flooded oil production wells. We found that *Arcobacter* and *Pseudomonas* were dominant both in the polymer-flooded and ASP-flooded production wells. *Halomonas* accounted for a large amount of the bacterial communities inhabiting in the ASP-flooded blocks, whereas they were hardly detected in the polymer-flooded blocks, and the trends for *Acetomicrobium* were the opposite. RDA analysis indicated that bacterial communities in ASP-flooded and polymer-flooded oil production wells are closely related to the physical and chemical properties, such as high salinity and strong alkaline, which together accounted for 56.91% of total variance. Co-occurrence network analysis revealed non-random combination patterns of bacterial composition from production wells of ASP-flooded and polymer-flooded blocks, and the ASP-flooded treatment decreased bacterial network complexity, suggesting that the application of ASP flooding technology reduced the tightness of bacterial interactions.

## Introduction

Several large oilfields in China, including Daqing Oil Field, have entered the late stage of high water cut production in recent years. The oil recovery of these oil reservoirs has been faced with different degrees of decline year by year. The average recovery ratio of crude oil is less than 30% by using the conventional oil recovery technology (Yang et al., 2004; Kang et al., 2011). Chemical enhanced oil recovery (EOR) technologies, including polymers flooding and alkaline/surfactant/polymer (ASP) flooding which were considered as the tertiary oil recovery have been widely used in many oil reservoirs (Chang et al., 2006). The mechanism of polymer flooding technology is to use hydrolyzed polyacrylamide to block the large channel in formation and improve the viscosity of displacement phase so as to improve the remaining oil recovery (Nasr-El-Din et al., 1991; Gao, 2013). ASP flooding technology could improve oil recovery by producing ultra-low interfacial tension at oil/water, improving oil fluidity, blocking large pore channels, and effectively improving the sweep efficiency of displacement phase (Wang et al., 2019). However, some polymers were adsorbed and trapped in the pores and surfaces of reservoir rocks, which made oil recovery more difficult. Improving oil recovery of the reservoirs after chemical flooding is a very important and arduous work. MEOR technology is a tertiary oil recovery method based on the activity of a microbial community producing oil-releasing metabolites (including biogas, biosurfactant, biomass, and acid) to enhance oil recovery (Van Hamme et al., 2003; Li et al., 2007; She et al., 2019; Nazina et al., 2020). Indigenous microorganisms have the advantages of strong adaptability to oil reservoir environment (Gao et al., 2018). Bacteria such as *Pseudomonas* sp., *Acinetobacter* sp., *Bacillus* sp., and *Rhodococcus* sp., etc., often occurring in oilfields, have the characteristics of degrading and/or emulsifying crude oil and reducing oil-water surface tension (Desai and Banat, 1997). It is very important to study the microbial community compositions and stimulation strategy in the process of MEOR with endogenous microbials (Xiao et al., 2013). However, the effects of polymer flooding and ASP flooding on indigenous bacterial communities are not understood in detail. Considering the critical roles of bacterial communities in MEOR process, revealing the influence of polymer flooding and ASP flooding on activity of bacteria could facilitate the implementation of MEOR technology in the future.

Due to the complexity of reservoir system (Pannekens et al., 2019) and the diversity of injection production technology, the ecosystem of the oil reservoirs appears to be more physiologically and phylogenetically complex and diverse than previously thought (Amend and Teske, 2005). In recent years, culture dependent and culture independent methods have been used to study microbial communities to reveal microbial diversity and genetic relationships inhabiting the oil reservoirs (Li et al., 2006). It has been reported that some microorganisms could metabolize polymers and low molecular weight polymer products (Gu et al., 1998). *Acinetobacter* sp., *Bacillus* sp., etc., isolated from oil reservoir environment were directly involved in the biodegradation of hydrolyzed polyacrylamide to reduce HPAM viscosity (Matsuoka et al., 2002; Youssef et al., 2009). However, to our knowledge, bacterial diversity and community compositions have rarely been reported in polymer-flooded and ASP-flooded reservoirs. *Pseudomonas* and *Acinetobacter* related with hydrocarbon biodegradation and surfactant production accounted for 17.79 and 6.02% in four produced wells of the reservoir after polymer flooding in Daqing Oil Field, respectively. However, these reports represented only a limited number of water samples from production wells in a separate block. Co-occurrence network analysis provides a deep understanding of the structure of microbial communities and the interactions between different microbes (Barberán et al., 2012). However, we have not found any reports about bacterial co-occurrence network patterns in the ASP-flooded and polymer-flooded reservoirs.

This study focused on comparison of the diversity and composition of bacterial communities and bacterial co-occurrence patterns in production waters from polymer-flooded and ASP-flooded blocks at the Daqing Oil Field. The injected alkali, surfactants, and hydrolyzed polyacrylamide that influenced the microbial communities in the ASP-flooded block have been detected. We hypothesize that (I) the community compositions of bacteria would be varied among polymer-flooded and ASP-flooded reservoirs. Alkali-tolerating populations in ASP-flooded blocks would be higher than those in polymer-flooded blocks, (II) the co-occurrence patterns of bacteria will differ from each other after the two EOR treatment. In conclusion, this study will enhance our understanding of bacterial community structure, interrelationships, and symbiotic networks in deep oil reservoir environment.

## Materials and Methods

### Samples Collection

The polymer-flooded blocks and ASP-flooded blocks are located in Daqing Oil Field in Northeast China. Both blocks had been developed by water flooding for several decades, and the average water content of the produced liquid is above 90%. Polymer flooding technology and ASP flooding technology were first applied in 1995 and 2014 in the Daqing Oil Field, respectively. The pH of the production water samples obtained from polymer-flooded blocks and ASP-flooded blocks were approximate 11.5 and 8.5, respectively, and the salinities were about 13,196 and 8,678 mg/L, respectively.

Water samples from 45 and 76 production wells at the polymer-flooded and ASP-flooded blocks, respectively, were collected into 10 L plastic containers. The containers were filled with oil-water production fluid to maintain an anoxic condition. All containers were autoclaved and rinsed with distilled water before sampling. About 1 L of water samples were centrifuged repeatedly under 12,000 g for 30 min to collect microbial cells (Pham et al., 2009; Tully et al., 2012). Genomic DNA was extracted from microbial cells using bacterial genomic extraction kit (TianGen, China). Quality of DNA was examined on agarose gel, and then used in the analysis described below or stored at −20°C.

### PCR Amplification and 16S rRNA Gene Sequencing

#### PCR Amplification

The V3-V4 hypervariable regions of the bacterial 16S rRNA gene were amplified with primers 338F (5′-ACTCCTACGGGAGGCAGCAG-3′) and 806R (5′-GGACTACHVGGGTWTCTAAT-3′) by thermocycler PCR instrument (Barberán et al., 2012). PCR reactions were performed in triplicate 20 μL mixture containing 12.5 μL of dNTP and Taq enzyme premix, 0.8 μL of each primer (5 μM) and 10 ng of template DNA. The PCR reactions were conducted by referring to the previous reference (Gloor et al., 2010). The PCR products were detected from a 2% agarose gel and further purified using the DNA Gel Extraction Kit (BoRi, China).

#### Illumina MiSeq Sequencing

The sequencing was performed on the Illumina MiSeq platform according to the standard protocols in Majorbiogroup, Shanghai, China.

#### Processing of Sequencing Data

Raw fastq files were demultiplexed using QIIME2, merged by FLASH and quality filtered by Trimmomatic to meet the following criteria: (I) The filtering of sequences with a tail quality score below 20 using a 50 bp sliding window; (II) barcodes and primers at both ends of the sequence were used to distinguish the samples, and the sequences with the maximum primer mismatch of two were removed; and (III) sequences whose minimum overlap length of 10 bp were merged according to the overlap relation between PE reads.

Operational taxonomic units (OTUs) were employed with 97% similarity using UPARSE. The representative sequence sets were aligned, and the taxonomy was analyzed by RDP Classifier algorithm^1^ against the Silva132 16S rRNA database at an 70% confidence level.

### Statistical Analysis

The relative OTU abundance was calculated as the percentage of sequences grouped into that OTU within a sample. The species richness of bacteria was counted by the numbers of OTUs in a sample. Whether the sequencing data volume was adequate or not was determined according to whether the species rarefaction curve reached a flat level. One-way ANOVA was performed to examine the alpha diversity among precipitation treatments, and the significant differences were determined with Duncan’s multiple comparison tests at the 95% confidence level. To explicitly test whether bacterial communities differed between two different EOR treatments, we first performed non-metric multidimensional scaling (NMDS) analysis for studying microorganisms of the oilfields. Next, we used PERMANOVA (1,000 permutations) using the adonis function of the VEGAN package in R on the OTU data matrix (Anderson, 2001; Jari Oksanen et al., 2016). Redundancy analysis (RDA) was performed in R to determine which environmental variables best explained the assemblage’s variability.

In order to analyze the effects of EOR on bacterial co-occurrence pattern, the underlying co-occurrences among bacterial taxa was depicted through network analysis. These OTUs with more than 20 sequences were retained for the construction of networks in order to reduce the complexity. Only robust (*r* > 0.6 or *r* < −0.6) and Significant Spearman correlations (*p* < 0.01) calculated within the “picante” R package (Xue et al., 2018) were incorporated into the network analyses (Hu et al., 2017). Network visualization and modular analysis were made with Gephi version 0.9.2. Node-level topological properties (i.e., degree, betweenness, closeness, and eigenector) were further calculated in the “igraph” R package. Statistical differences in measured node-level attributes across different taxa were determined using non-parametric Mann–Whitney *U*-test. Nodes with high degree (in top 1%) are recognized as keystone species in co-occurrence networks (Marasco et al., 2018).

## Results

### Overall Pyrosequencing Information

Using Illumina MiSeq sequencing, 4460334 16S rRNA sequences ranging in length from 200 to 592 bp (most of the sequences were 435 bp) from 76 production water samples at ASP-flooded blocks and 2181316 16S rRNA sequences ranging from 167 to 592 bp (most of the sequences were 434 bp) from 45 production water samples at polymer-flooded blocks were obtained. A total of 1,451 OTUs were detected in 16S rRNA libraries constructed from production water samples obtained at polymer-flooded and ASP-flooded blocks based on 97% similarity (Supplementary Table S1). These OTUs belonged to 48 phyla, 76 classes, 302 families, 603 genera, and 935 species. The species rarefaction curve reached saturation with increasing sample numbers, indicating that the sequencing data volume had reached the requirements of sequencing (Supplementary Figure S1). The diversity (Simpson and Shannon) and richness (ACE and Chao 1) indices for the OTUs from bacterial communities were determined for all samples (Supplementary Table S2). The results showed that there was no significant difference in the diversity and richness of bacterial communities between polymer-flooded blocks and ASP-flooded blocks. Our results indicated that the two EOR technologies did not cause significant differences in the diversity and richness of the bacterial communities in the production wells.

### Bacterial Community Compositions in Polymer-Flooded and ASP-Flooded Blocks

**FIGURE 1:**
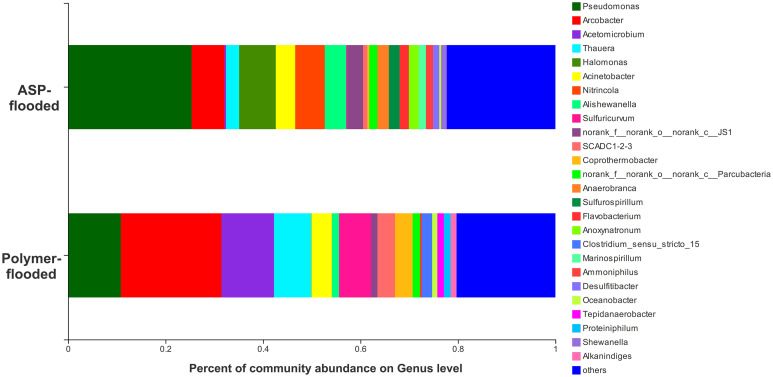
Bacterial community compositions at the genus level from production wells at ASP-flooded and polymer-flooded blocks.

**FIGURE 2:**
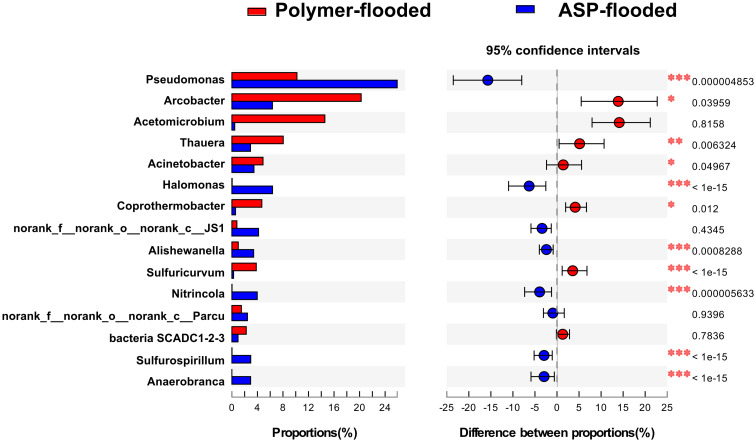
Wilcoxon rank-sum test revealed the bacterial populations with significant differences in the relative abundance between ASP-flooded blocks and polymer-flooded blocks. ^∗^*P* < 0.05, ^∗∗^*P* < 0.01, ^∗∗∗^*P* < 0.001.

**FIGURE 3:**
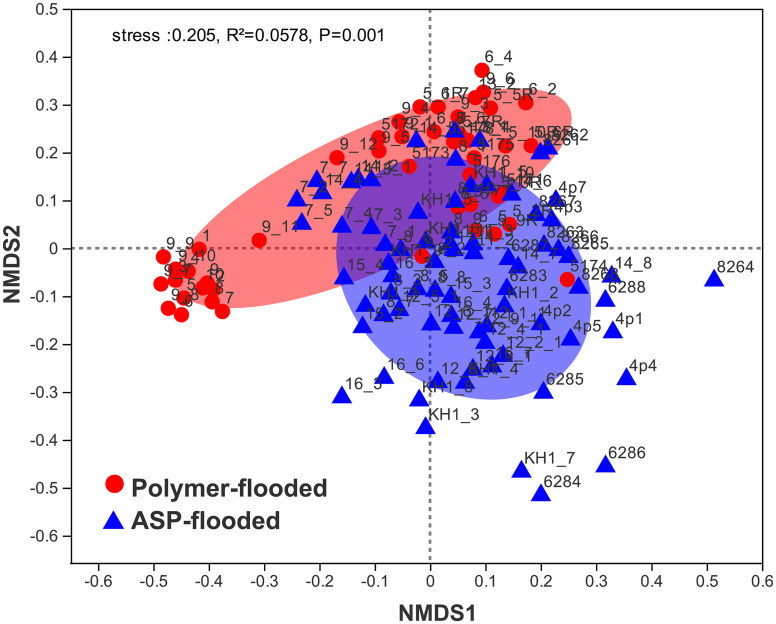
Non-metric multidimensional scaling (NMDS) ordination plot of bacterial communities from production wells at ASP-flooded and polymer-flooded blocks. PERMANOVA analysis showed significant differences in bacterial communities between ASP-flooded blocks and polymer-flooded blocks.

Four phyla including *Proteobacteria*, *Epsilonbacteraeota*, *Firmicutes*, and *Synergistetes* were dominant (relative abundance > 5%), accounting for 84.33% of the total bacteria in the libraries from production wells at polymer-flooded blocks. In contrast, three phyla including *Proteobacteria*, *Epsilonbacteraeota*, and *Firmicutes* were dominant (relative abundance > 5%), accounting for 85.52% of the total bacteria in production wells at polymer-flooded blocks (Supplementary Figure S2). At the genus level, *Arcobacter*, *Pseudomonas*, *Acetomicrobium*, *Thauera*, and *Sulfuricurvum* were dominant (relative abundance > 5%), accounting for 56.47% of the total bacteria in polymer-flooded production wells. Bacteria of the genera *Pseudomonas*, *Halomonas*, *Arcobacter*, and *Nitrincola* were dominant, accounting for 45.68% in ASP-flooded production wells (Figure 1). At the phylum level, a Wilcoxon rank-sum test indicated that *Proteobacteria*, *Synergistetes*, *Bacteroidetes*, *Atribacteria, Coprothermobacteraeota*, *Thermotogae*, and *Caldiserica* were the dominant populations, the relative abundances difference was significant between production wells at polymer-flooded and ASP-flooded blocks (Supplementary Figure S3). *Proteobacteria* was the predominant group in production wells at polymer-flooded and ASP-flooded blocks. Relative abundance of *Proteobacteria* was higher in production water samples from polymer-flooded blocks than from ASP-flooded blocks. At the genus level, a Wilcoxon rank-sum test indicated that *Pseudomonas*, *Thauera*, *Acinetobacter*, and *Halomonas* were the dominant populations with significantly different relative abundances between the polymer-flooded block and ASP-flooded block (Figure 2). The relative abundance of *Acinetobacter* was higher in production wells at polymer-flooded blocks than in production wells at ASP-flooded blocks. NMDS analysis based on relative abundance of OTUs showed differences in bacterial community compositions between polymer-flooded blocks and ASP-flooded blocks (Figure 3). This observation was confirmed by multivariate analysis PERMANOVA (*R*^2^ = 0.058, *P* = 0.001).

### Bacterial Co-occurrence Network Analysis

**FIGURE 4:**
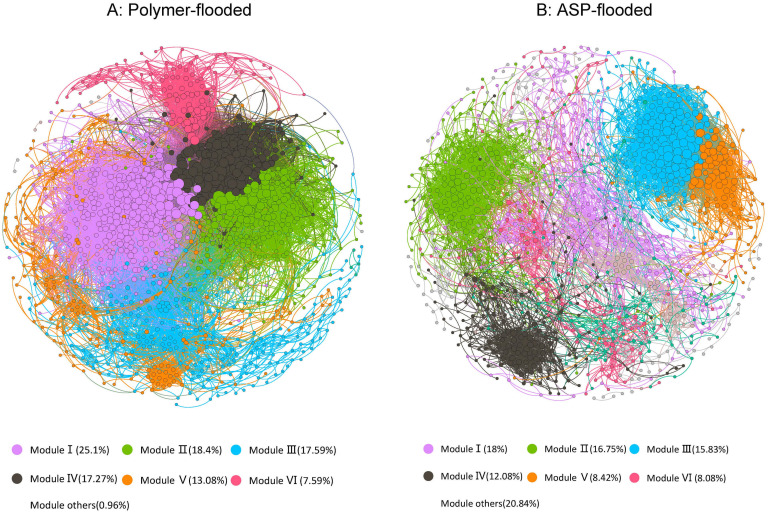
The co-occurrence patterns among OTUs revealed by network analysis. The nodes were colored according to different types of modularity classes **(A)** and supergroups **(B)**, respectively. A connection stands for a strong (Spearman’s *r* > 0.6 or *r* < –0.6) and significant (*P*-value < 0.01) correlation.

In order to determine the general effects of Enhanced oil recovery (EOR) treatments on bacterial associations, two networks were constructed for two enhanced oil recovery practices by combining all bacteria originating from polymer-flooded and ASP-flooded blocks (Figure 4). The modularity index (MD) was 0.655 and 0.483 in network of polymer-flooded and ASP-flooded blocks, respectively, where *MD* > 0.4 suggests that the network has a modular structure, indicating that the network structure was non-random (Xue et al., 2017). The polymer-flooded blocks’ network consisted of 1,242 nodes linked by 39,614 edges, and the ASP-flooded blocks’ network consisted of only 1,200 nodes with 10,073 edges. Compared to polymer flooding treatment, the clustering coefficient of the network of ASP-flooded blocks was decreased by 0.040, and the network density of microbiome networks in polymer-flooded blocks was decreased by 0.037, indicating that oil reservoir bacterial associations were more tightened in responding to the application of polymer flooding technology.

In the co-occurrence network, *Proteobacteria*, *Acidobacteria*, and *Firmicutes* (Genus-level classifications are presented in Supplementary Materials) appeared most frequently, indicating that these generalists had adapted to these environments. Keystone taxa were defined as taxa interacting with many other members (i.e., top 1% of interactions), which were thought to play crucial roles in the overall community (Berry and Widder, 2014). A total of 12 OTUs were defined as keystone species in polymer-flooded blocks’ network, including *Proteobacteria* (2 OTUs), *Firmicutes* (2 OTUs), *Chloroflexi* (2 OTUs), *Caldiserica* (1 OTU), *Bacteroidetes* (1 OTU), *Actinobacteria* (1 OTU), *Synergistetes* (1 OTU), and unclassified Bacteria (2 OTUs). There also 12 keystone species were defined in ASP-flooded blocks’ network, including *Firmicutes* (5 OTUs), *Patescibacteria* (2 OTUs), *Actinobacteria* (1 OTU), Chloroflexi (1 OTU), *Verrucomicrobia* (1 OTU), and unclassified bacteria (2 OTUs).

### Factors Related to Variation of the Bacterial Community

**FIGURE 5:**
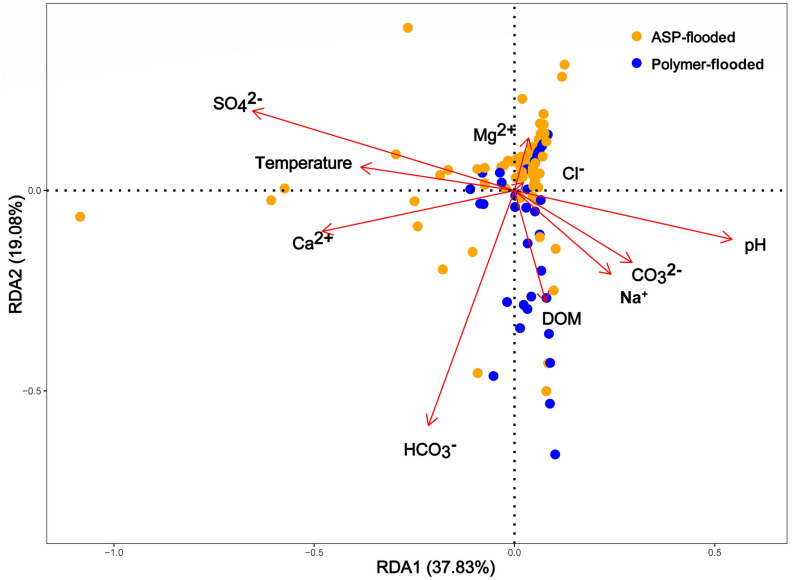
Spatially constrained, distance-based redundancy analysis of plot based quantitative bacterial community composition. The ASP-flooded and polymer-flooded samples (different color) are shown. Arrows indicate the direction of the maximum change in variables. DOM, Degree of mineralization (total salinity of water).

Physicochemical parameters of production water samples, such as CO_3_^2–^, pH, HCO_3_^2–^, Mg^2+^, Ca^2+^, Na^+^, and temperature were all significantly different among polymer flooded and ASP-flooded blocks (Supplementary Table S3). In order to explore the relationship between environmental factors and the microbial community in different water-flooded oil reservoirs, Redundancy Analyses (RDA) were conducted. Based on the results of the RDA (Figure 5), there were large variations between polymer-flooded and ASP-flooded blocks, the first two RDA axes explained a total of 56.91% of the variance in the composition of bacterial communities among the two EOR treatments. pH (*R*^2^ = 0.12, *p* = 0.002), temperature (*R*^2^ = 0.06, *p* = 0.037), SO_4_^2–^ (*R*^2^ = 0.17, *p* = 0.007), HCO_3_^–^ (*R*^2^ = 0.11, *p* = 0.002), and Ca^2+^ (*R*^2^ = 0.09, *p* = 0.007) showed strongly significant correlation to the species of distribution of bacterial communities.

## Discussion

In this study, we examined the bacterial community assembly in oil reservoir after application of different EOR technologies. Our results showed that *Proteobacteria*, *Epsilonbacteraeota*, *Firmicutes*, and *Synergistetes* were predominant in the total sequences in both studied sites. *Arcobacter*, *Pseudomonas*, *Acetomicrobium*, *Thauera*, and *Sulfuricurvum* were dominant (relative abundance > 5%) in polymer-flooded production wells. *Pseudomonas*, *Halomonas*, *Arcobacter*, and *Nitrincola* were dominant in ASP-flooded production wells. These results suggested that the bacterial community structure was significantly different among polymer-flooded and ASP-flooded blocks, whereas bacterial diversity was not affected by different EORs. This is in accordance with the previous investigation on microbial communities in oil reservoirs, which showed that bacterial community was significantly influenced by reservoir environments (Gao et al., 2016, 2019). The injected alkali, surfactants, and hydrolyzed polyacrylamide have greatly affected the microbial communities in ASP-flooded blocks. We found that the environmental changes that occur with changes in EOR process contribute differently to bacterial community. In particular, in our research, the injected alkali could result in the pH increase of formation water up to 10.76 in ASP-flooded blocks, which is much higher than those of polymer flooded blocks with pH 8.2 (Supplementary Table S3). The results showed the alkaline environment may have served as a major selective force in ASP-flooded blocks. The high pH inhibited the survival of most microbial populations. Recent studies have also demonstrated that bacterial communities were strongly correlated with environmental pH (Fierer and Jackson, 2006; Rousk et al., 2010). Alkali-tolerating populations *Pseudomonas*, *Halomonas*, *Alishewanella*, *Sulfurospirillum*, *Nitrincola*, and *Anaerobranca* in ASP-flooded blocks were higher than those in polymer-flooded blocks. Besides pH, CO_3_^2–^, HCO_3_^–^, Ca^2+^, and Na^+^ could also be important driving factors for shaping bacterial communities in the oil reservoirs.

In the study, we found that some functional microbes which could enhance oil production are dominant in polymer-flooded and ASP-flooded reservoirs. Proteobacteria dominated in both polymer-flooded and ASP-flooded blocks. Relative abundance was higher in ASP-flooded blocks than in polymer-flooded blocks. *Pseudomonas* sp., which belongs to *Proteobacteria*, has been widely used in tertiary oil recovery and pollution remediation due to its capability to degrade petroleum hydrocarbons, produce surfactants, and degrade polyacrylamide (Banat, 1995). In addition, the rhamnolipid produced by *Pseudomonas* sp. could reduce the interfacial tension of oil-water phase (Bognolo, 1999) to EOR (Chrzanowski et al., 2012). *Acinetobacter* sp. were also found in both polymer-flooded and ASP-flooded blocks, and the relative abundance was higher in polymer-flooded blocks than in ASP-flooded blocks. *Acinetobacte*r sp. has the ability to degrade saturated and aromatic hydrocarbons (Sugiura et al., 1996). These results indicate that both the polymer-flooded and ASP-flooded reservoirs have the basis of implementing microbial oil recovery technologies.

Networks analysis could perform richness and composition analysis and could also provide in-depth research on microbial interaction and ecological assembly rules (Ziegler et al., 2018). In the study, for the first time we applied correlation-based network analysis to explore the co-occurrence models of bacterial communities across polymer-flooded and ASP-flooded blocks. Previous studies have shown that microbial interactions can maintain ecosystem function and stability (Ziegler et al., 2018). Our results showed that network complexity was higher in polymer-flooded blocks than in ASP-flooded blocks. Increased network connectivity and complexity are previously undescribed properties of bacterial community assemblages and represent fundamental differences microhabitat among polymer-flooded and ASP-flooded blocks. Our results also showed that fewer connections and lower clustering coefficient and network density were observed in co-occurrence network of ASP-flooded blocks when compared with those of polymer-flooded blocks. The increase of pH of formation water resulted in the reduction of the most connections (Nearly 3/4) in the ASP-flooded blocks’ network. This may also indicate that the alkaline environment served as a major selective force in ASP-flooded blocks. Keystone players were thought to play crucial roles in the overall microbiome and could maintain the network stability and structure (Berry and Widder, 2014). *Firmicutes* and *Patescibacteria* were more important in ASP-flooded blocks than in polymer-flooded blocks, this suggested that they may play an irreplaceable role in maintaining the structure of bacterial communities in high pH environments. Previous studies have shown that microbial interactions can maintain ecosystem function and stability (Ziegler et al., 2018).

## Conclusion

In this paper, the characteristics of bacterial communities in ASP-flooded blocks were revealed by comparison with those of polymer-flooded blocks. We found that application of different EOR technologies significantly altered bacterial community compositions but had no remarkable effects on species diversity and richness. Bacteria of the genera *Arcobacter*, *Pseudomonas*, *Acetomicrobium*, *Thauera*, and *Sulfuricurvum* were dominant in formation water samples from polymer-flooded blocks. The extreme environment of the ASP-flooded reservoirs led to the predominance of alkali-tolerating *Pseudomonas*, *Halomonas*, and *Nitrincola*. The network of bacterial communities at the ASP-flooded blocks was less complex and tighter than that of polymer-flooded blocks. Indigenous microbes are better adapted to the oil reservoir environment, and how to stimulate these bacteria to enhance oil recovery would be the basis for meaningful future work.

## Data Availability Statement

The original contributions presented in the study are publicly available. This data can be found here: https://www.ncbi.nlm.nih.gov/sra/PRJNA673922.

## Author Contributions

JLW, and GLR proposed and organized the overall project. MH, WL, LHB, and LNQ performed the majority of the experiments. MH and LNQ gave assistance in lab work and laboratory analyses. JLW and GLR wrote the main manuscript text. YTZ, JJL, XMD, YL, LLB, XHC and contributed insightful discussions. All authors reviewed the manuscript.

## Conflict of Interest

WL, JJL, XMD, and XHC were employed by company Daqing Oil Field Company, Ltd. The remaining authors declare that the research was conducted in the absence of any commercial or financial relationships that could be construed as a potential conflict of interest.

## Footnotes

1 http://rdp.cme.msu.edu/
