# Supplementary material for: Compositions and Co-occurrence Patterns of Bacterial Communities Associated With Polymer- and ASP-Flooded Petroleum Reservoir Blocks
Source: Front Microbiol. 2020 Dec 1;11:580363. doi: 10.3389/fmicb.2020.580363 (PMC7736161; doi:10.3389/fmicb.2020.580363)
Supplement: Supplementary file 1 [file Data_Sheet_1.PDF]

Table S1 The rarefied sample OTU matrix. See supplementary materials excel file.

Table S2 The diversity of bacteria in each sample. See supplementary materials excel file.

Table S3. Physical and chemical characteristics (mean±SE) of samples collected from different EOR treatment. Data with different lowercase letters and “*” indicate significant levels at $P < 0.05$.

	pH	CO_3^{2-} (g/L)	HCO_3^- (g/L)	Tempe -rature	Cl^- (g/L)	SO_4^{2-} (g/L)	Mg^{2+} (mg/L)	Na^+ (g/L)	DOM (g/L)	Ca^{2+}
Polymer	8.20b	0.37b	3.51a	46.46a	1.10a	0.13a	10.39b	1.93b	7.26a	27.11b
ASP	10.76a	1.89a	3.35a	46.19b	0.96a	0.07a	10.85a	3.52a	6.63a	54.53a

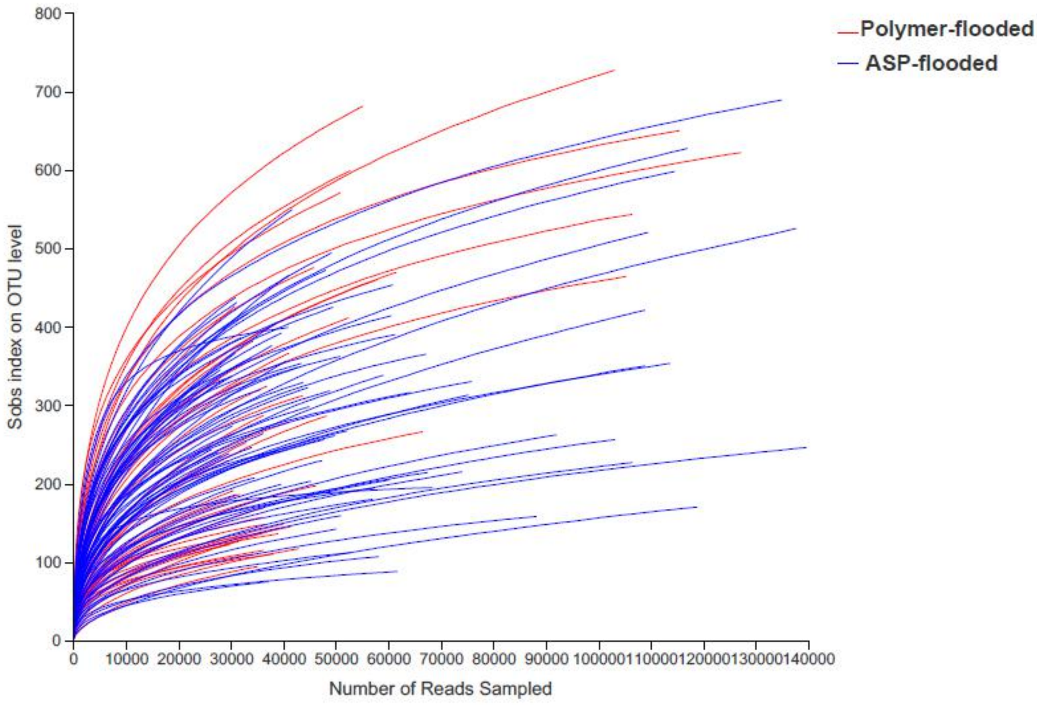

Figure S1 | Taxa accumulation curves of bacteria in all polymer-flooded and ASP-flooded production samples.

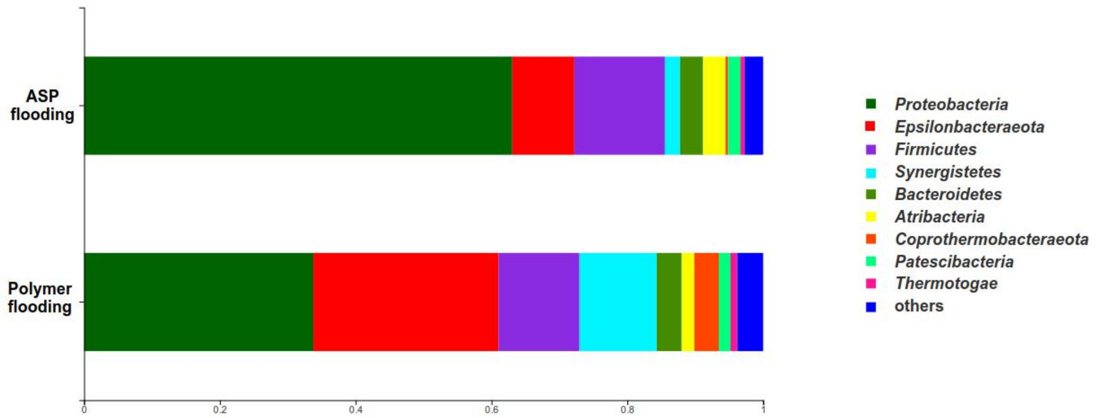

Figure S2 | Bacterial community compositions at phylum level in the ASP flooding production wells and polymer flooding production wells.

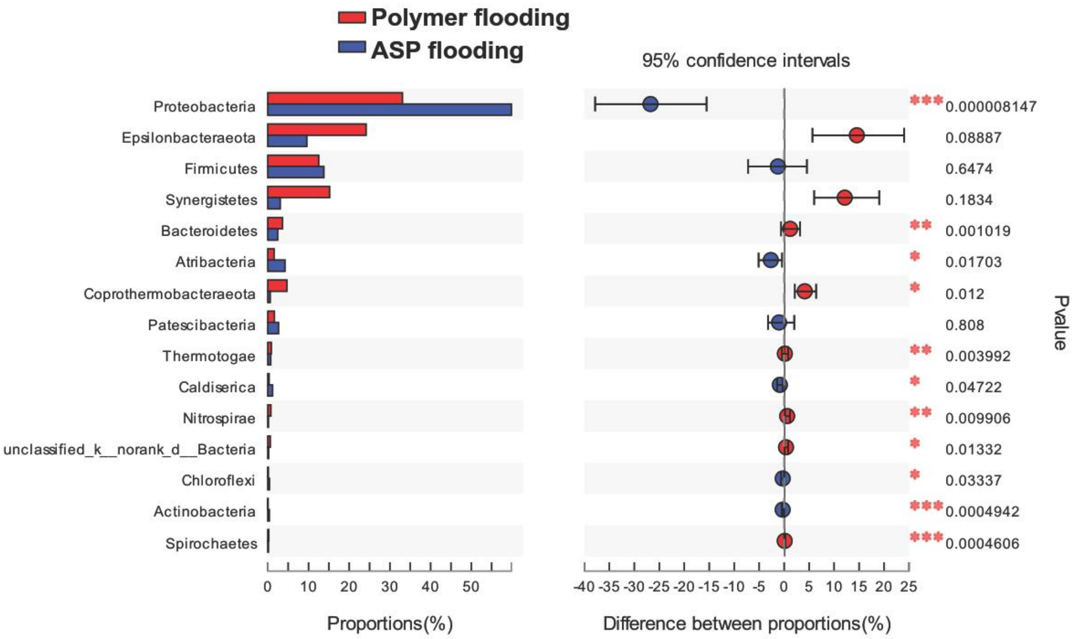

Figure S3 | Wilcoxon rank-sum test revealed the bacterial populations with significant differences in the relative abundance between the ASP flooding wells and polymer flooding wells at phylum level. * represent P < 0.05, ** represent P < 0.01, *** represent P < 0.001.
